# Editorial Comment: Thulium fibre laser versus Holmium:YAG for ureteroscopic lithotripsy: outcomes from a prospective randomised clinical trial

**DOI:** 10.1590/S1677-5538.IBJU.2023.02.03

**Published:** 2023-05-29

**Authors:** Alexandre Danilovic

**Affiliations:** 1 Hospital das Clinicas Faculdade de Medicina Universidade de São Paulo São Paulo SP Brasil Departamento de Urologia, Hospital das Clinicas Faculdade de Medicina da Universidade de São Paulo – FMUSP, São Paulo, SP, Brasil;; 2 Hospital Alemão Oswaldo Cruz São Paulo SP Brasil Serviço de Urologia, Hospital Alemão Oswaldo Cruz, São Paulo, SP, Brasil

Øyvind Ulvik ^1^, Mathias Sørstrand Æsøy ^2^, Patrick Juliebø-Jones ^3^, Peder Gjengstø ^2^, Christian Beisland ^3^ *Eur Urol . 2022 Jul;82(1):73-79.*

## COMMENT

This randomized controlled trial is the first to compare the outcomes after ureterorenoscopic lithotripsy (URS) with low power Holmium:YAG (Ho:YAG) and Thulium Fibre Laser (TFL). Primary endpoint was the stone-free rate (SFR) by computed tomography (CT) at 3 months after URS. Secondary endpoints were the operative time and complications ( 1 ).

This is a very important paper since the authors demonstrated for the first time a superiority of TFL over Ho:YAG for the treatment of renal stones. Overall higher SFR was achieved with TFL (57% vs. 80%, p=0.006). Ureteral stones SFR was 100% in both groups but renal stones SFR was 33% for Ho:YAG vs. 66% for TFL (p=0.005). Operative time was shorter with TFL (49 min) than with Ho:YAG (57 min). There was no difference in readmissions between groups (12% TFL vs. 13% Ho:YAG, p=1) and no ureteral strictures or hydronephrosis were observed on 3-month CT ( 1 ).

The reported 33% SFR for renal stones of Ho:YAG arm and 66% for TFL are very disappointing. The SFR reported by Ulvik et al. is comparable to the SFR of shockwave lithotripsy (SWL) reported by Bosio et al. in their randomized controlled trial of URS vs. SWL and to the 34.1% SFR of SWL evaluated by CT of the prospective study by Torricelli et al. ( 2 , 3 ). Also, Ulvik et al. results are inferior to many previously reported studies using low power Ho:YAG as the 74.8% SFR evaluated by 3-months CT using basketing strategy and ureteral access sheath (UAS) in every case ( 4 ). The facts that UAS was not used in any case and that nine different (three faculty and six residents) surgeons performed 120 URS (mean of 13.3 procedures/surgeon) could help to explain low SFR. Of note, the authors decided to start laser settings in 0.4J/6Hz and limit laser settings to 0.8J/20Hz in renal pelvis. These settings are more consistent with basketing strategy. Despite there is no definitive evidence for superiority of dusting versus basketing, the later requires more operative time to achieve better SFT ( 5 ). Since the authors recommend low laser settings, it would be interesting to use basketing more efficiently to increase their SFR for both Ho:YAG and TFL.

Other groups should report their experience with TFL in randomized controlled trial to confirm TFL superiority over Ho:YAG in different scenarios.
